# Reduced Risk of Hepatocellular Carcinoma in Patients with Chronic Hepatitis B Receiving Long-Term Besifovir Therapy

**DOI:** 10.3390/cancers16050887

**Published:** 2024-02-22

**Authors:** Hyung Joon Yim, Seong Hee Kang, Young Kul Jung, Sang Hoon Ahn, Won Kim, Jin Mo Yang, Jae Young Jang, Yong Oh Kweon, Yong Kyun Cho, Yoon Jun Kim, Gun Young Hong, Dong Joon Kim, Joo Hyun Sohn, Jin Woo Lee, Sung Jae Park, Sun Young Yim, Jin Kyung Park, Soon Ho Um

**Affiliations:** 1 Department of Internal Medicine, Korea University Ansan Hospital, 123, Jeokgeum-ro, Danwon-gu, Ansan 15355, Republic of Korea; dumbo83@korea.ac.kr (S.H.K.); 2002021168@korea.ac.kr (Y.K.J.); 2 Department of Internal Medicine, Severance Hospital, Yonsei University College of Medicine, 50-1, Yonsei-ro, Seodaemun-gu, Seoul 03722, Republic of Korea; ahnsh@yuhs.ac; 3 Department of Internal Medicine, Seoul Metropolitan Government Seoul National University Boramae Medical Center, 20 Boramae-ro 5-gil, Dongjak-gu, Seoul 07061, Republic of Korea; drwon1@snu.ac.kr; 4 Department of Internal Medicine, St. Vincent’s Hospital, College of Medicine, The Catholic University of Korea, 93 Jungbu-daero, Paldal-gu, Suwon 16247, Republic of Korea; jmyangdr@catholic.ac.kr; 5 Department of Internal Medicine, Soonchunhyang University Seoul Hospital, 59, Daesagwan-ro, Yongsan-gu, Seoul 04401, Republic of Korea; jyjang@schmc.ac.kr; 6 Department of Internal Medicine, Kyungpook National University Hospital, 680 gukchaebosang-ro, Jung-gu, Daegu 41944, Republic of Korea; yokweon@knu.ac.kr; 7 Department of Internal Medicine, Kangbuk Samsung Hospital, Sungkyunkwan University School of Medicine, 29 Saemunan-ro, Jongno-gu, Seoul 03181, Republic of Korea; choyk2004.cho@samsung.com; 8 Department of Internal Medicine and Liver Research Institute, Seoul National University Hospital, 101 Daehak-ro, Jongno-gu, Seoul 03080, Republic of Korea; yoonjun@snu.ac.kr; 9 Department of Internal Medicine, Kwangju Christian Hospital, 37 Yangnim-ro, Nam-gu, Gwangju 61661, Republic of Korea; gyh228803@gmail.com; 10 Department of Internal Medicine and Center for Liver and Digestive Diseases, Hallym University Chuncheon Sacred Heart Hospital, 77 Sakju-ro, Chuncheon 24253, Republic of Korea; djkim@hallym.ac.kr; 11 Department of Internal Medicine, Hanyang University Guri Hospital, 153, Gyeongchun-ro, Guri-si 11923, Republic of Korea; sonjh@hanyang.ac.kr; 12 Department of Internal Medicine, Inha University Hospital, 27 Inhang-ro, Jung-gu, Incheon 22332, Republic of Korea; jin@inha.ac.kr; 13 Department of Internal Medicine, Inje University Busan Paik Hospital, 75 Bokji-ro, Busanjin-gu, Busan 47392, Republic of Korea; yaheaven@inje.ac.kr; 14 Department of Internal Medicine, Korea University Anam Hospital, 73 Goryeodae-ro, Seongbuk-gu, Seoul 02841, Republic of Korea; eug203@korea.ac.kr; 15 Ildong Pharmaceutical Company, 2, Baumoe-ro 27-gil, Seocho-gu, Seoul 06752, Republic of Korea; jkpark@ildong.com

**Keywords:** antivirals, cancer, carcinogenesis, complications, hepatitis B virus, liver tumor, nucleotide analogues, performance, prediction model, risk reduction

## Abstract

**Simple Summary:**

Further information is necessary regarding the influence of besifovir (BSV), a new nucleotide analogue, on the occurrence of hepatocellular carcinoma (HCC) in patients with chronic hepatitis B (CHB). When we compared the HCC incidence in non-cirrhotic CHB patients receiving BSV with the predicted number derived from the REACH-B (risk estimation for HCC in CHB) model, the standardized incidence ratio (SIR) was significantly reduced to 0.128 at 7 years. The incidence of HCC in patients with cirrhosis was compared using the GAG-HCC (guide with age, gender, HBV DNA, core promotor mutation, and cirrhosis) model, and the SIR was significantly decreased to 0.371 at 7.5 years. HCC prediction was available for BSV-treated patients using existing models. We concluded that BSV decreases the risk of HCC in patients with CHB, and HCC risk prediction models are applicable.

**Abstract:**

No information is available regarding the influence of besifovir (BSV), a new nucleotide analogue, on the occurrence of hepatocellular carcinoma (HCC) in patients with chronic hepatitis B (CHB). This study evaluated the reduced risk of HCC in patients undergoing BSV treatment. A total of 188 patients with CHB were treated with BSV for up to 8 years. We prospectively assessed the incidence of HCC compared with the risk from prediction models. During the follow-up, 5 patients developed HCC: 1 of 139 patients with non-cirrhotic CHB, and 4 of 49 patients with liver cirrhosis. We compared the HCC incidence in non-cirrhotic and cirrhotic patients with the predicted number derived from the REACH-B (risk estimation for HCC in CHB) model and GAG-HCC (guide with age, gender, HBV DNA, core promotor mutation, and cirrhosis) model, respectively. The standardized incidence ratio (SIR) was 0.128 (*p* = 0.039) at 7 years in non-cirrhotic CHB patients, and the SIR was 0.371 (*p* = 0.047) at 7.5 years in cirrhotic patients, suggesting a significantly decreased HCC incidence in both groups. HCC prediction was available for BSV-treated patients using existing models. In conclusion, BSV decreased the risk of HCC in patients with CHB, and prediction models were applicable. Clinical trial registry website and trial number: ClinicalTrials.gov no: NCT01937806.

## 1. Introduction

Chronic hepatitis B virus (HBV) infection is a major risk factor for the development of hepatocellular carcinoma (HCC) [1]. To ensure adequate HCC surveillance, several risk prediction models for HCC have been developed for patients with chronic HBV infection. Initially, prediction models such as GAG-HCC (guide with age, gender, HBV DNA, core promotor mutation, and cirrhosis) [2], CU-HCC (Chinese University HCC) [3], and REACH-B (risk estimation for HCC in chronic hepatitis B) were developed for treatment-naïve chronic hepatitis B (CHB) patients with or without liver cirrhosis [4]. More recently, additional models have been devised for patients receiving antiviral therapies, especially entecavir (ETV) or tenofovir disoproxil fumarate (TDF) [5,6,7], since it has been reported that long-term viral suppression reduces the risk of HCCs [8,9]. The degree of risk reduction was reported to be comparable between ETV and TDF treatment, although there is still a debate [10,11].

Besifovir dipivoxil maleate (BSV) is a new potent antiviral agent approved in Korea [12]. The previous phase 3 clinical trial demonstrated the similar antiviral efficacy of BSV and TDF in the treatment-naïve CHB patients [12]. Moreover, BSV therapy was associated with a lower incidence of renal injury and bone damage compared with TDF [12]. Hence, it is recommended as one of the first-line agents for the treatment of CHB in Korea [13]. In addition, a recent report suggested that treatment with BSV improves hepatic histology and decreases intrahepatic covalently closed circular DNA (cccDNA) [14]. Considering that hepatic necroinflammation, liver fibrosis, cirrhosis, and the persistence of cccDNA are associated with the development of HCC [1,15], the favorable effect of BSV on these factors may lower the risk of HCC in patients with CHB. However, to date, there are no data for the influence of BSV treatment on the occurrence of HCC.

The present study aimed to assess the degree of reduction in HCC incidence under the BSV treatment compared to predictive numbers and to evaluate the current HCC risk prediction models that are the best fit for CHB patients receiving long-term BSV therapy.

## 2. Materials and Methods

### 2.1. Patients

The present study is a part of an extensional phase 3 trial for the safety and efficacy of BSV [12]. The inclusion and exclusion criteria were previously reported [12]. Briefly, treatment-naïve CHB patients over 20 years old with HBV DNA levels > 17,241 IU/mL for hepatitis B e antigen (HBeAg)-positive or >1724 IU/mL for HBeAg-negative were enrolled. Patients with elevated alpha-fetoprotein and suspicion of HCC on computed tomography or previous history of HCC were excluded. Patients were randomly assigned to the BSV 150 mg (Ildong Pharmaceutical, Seoul, Republic of Korea) or TDF 300 mg (Gilead Sciences, Foster City, CA, USA) groups and received the drugs for 48 weeks. After 48 weeks, all patients were treated with BSV for up to 384 weeks and followed for up to 396 weeks for safety analysis (8 years). During BSV therapy, 660 mg of L-carnitine was concurrently administered as a supplement. Other nucleos(t)ide analogues or interferons were not allowed by the protocol. We assessed the incidence of HCC during follow-up.

This clinical trial was approved by the institutional review board at each site (approval no. I1306111 at Korea University Ansan Hospital) and conducted in accordance with the International Conference on Harmonization Guidelines for Good Clinical Practice and the Declaration of Helsinki. Written informed consent was obtained from all participants.

### 2.2. Primary and Secondary Endpoints

The primary endpoint of the study was the observed incidence of HCC compared with the predictive number of HCC prediction models derived from untreated CHB patients. The secondary endpoints included the performance of current models for HCC prediction under antiviral therapy in a cohort of patients with CHB receiving BSV therapy. We also evaluated the long-term antiviral efficacy and antifibrotic effects of BSV before the assessment of HCC models.

### 2.3. Clinical Assessment and Definitions

During the follow-up of 8 years, routine laboratory tests were performed every 12 weeks, and serum alpha-fetoprotein and liver ultrasonography were performed every 24 weeks for HCC surveillance. HBV DNA was quantified using the COBAS AmpliPrep/TaqMan test (Roche Diagnostics, Indianapolis, IN, USA) with a lower detection limit of 20 IU/mL. Hepatitis B e antigen (HBeAg) or hepatitis B surface antigen (HBsAg) were tested with a standard assay, as reported previously [12]. Noninvasive fibrosis markers, such as aspartate aminotransferase-to-platelet ratio indexes (APRI) and Fib-4, were estimated annually to assess liver fibrosis [12].

Chronic HBV infection was defined as detection of HBsAg for more than 6 months. Liver cirrhosis was defined by histological and clinical criteria: (1) microscopic findings of liver cirrhosis (F5 or F6) using the Ishak–Knodell scoring system; (2) ultrasonography findings suggestive of cirrhosis, including a blunted edge, nodular liver surface, and splenomegaly; or (3) liver fibrosis biomarkers predictive of liver cirrhosis (≥Fib-4 score 3.6). HCC was diagnosed using histological or clinical criteria according to the guidelines of the European Association for the Study of the Liver [16]. Diabetes mellitus (DM) was considered based on fasting glucose levels > 126 mg/dL, hemoglobin A1c > 6.5%, or concomitant use of anti-diabetic medications.

### 2.4. Models for the Prediction of HCCs

The variables and characteristics of the HCC prediction models utilized in this study are summarized in Table S1: The GAG-HCC and REACH-B models were developed for HCC prediction in treatment-naïve patients with CHB. The PAGE-B (platelet count, age, and gender) [5], mPAGE-B (modified PAGE-B) [17], CAMD (cirrhosis, age, male sex, and DM) [18], HCC-RESCUE (HCC-risk estimating score in CHB under ETV) [6], REAL-B (real-world effectiveness from the Asia Pacific rim liver consortium for HBV) [19], and AASL (age, albumin, sex, and liver cirrhosis)-HCC models were developed in patients receiving ETV or TDF [20]. In this study, we did not include models using liver stiffness measurement values, which were not collected during the clinical trial. Additionally, HCC prediction models developed not only from CHB but also from various etiologies of chronic liver diseases were excluded (Table S1).

First, we compared the observed incidence of HCC with the predictive number using the REACH-B or GAG-HCC models. We then evaluated the performance of the other models for the prediction of HCC in patients with CHB under long-term BSV treatment.

### 2.5. Statistical Analysis

The baseline characteristics of the patients and their laboratory data were compared using the independent *t*-test or Wilcoxon rank sum test, as appropriate, depending on the results of the normality test for continuous variables, and the results are expressed as means ± standard deviations. For categorical variables, the chi-square test or Fisher’s exact test was performed as appropriate, and the proportions of the patients in each group were expressed as percentages.

We assessed the cumulative incidence of virological/biochemical response and HCC using the Kaplan–Meier method and compared them using the log-rank test between the patients with and those without liver cirrhosis. The overall changes in noninvasive fibrosis scores were compared between the groups using a linear mixed model for repeated measurements. To minimize the preceding hepatocarcinogenic effects of chronic HBV infection, patients with HCC that developed within 12 months of enrollment were excluded from the study. We calculated the standardized incidence ratio (SIR) based on the predictive incidence of HCC from the REACH-B and GAG-HCC models in non-cirrhotic patients and those with liver cirrhosis, respectively. Disease-free survival probability for the estimation of the annual incidence of HCC was provided by Yang et al., who proposed the REACH-B model [12].

To evaluate the risk factors for development of HCC, we performed univariable and multivariable analyses using Cox’s proportional hazards regression model.

The performance of the HCC prediction models was evaluated using the area under the receiver operating characteristic curve (AUROC) analysis, and the values were compared using the DeLong test. All statistical analyses were performed using the SAS software (version 9.4; SAS Institute, Cary, NC, USA), and *p*-values < 0.05 was considered statistically significant.

## 3. Results

### 3.1. Baseline Characteristics of the Patients

A total of 188 patients with chronic HBV infection were enrolled in this study (Figure S1). Among them, 139 patients did not have underlying liver cirrhosis and 49 had liver cirrhosis at baseline. There were differences between the groups in terms of age, platelet counts, serum albumin, and alpha-fetoprotein levels. A seven-year-older mean age, approximately 20% higher prevalence of DM, 50,000/μL lower platelet count, and 26 ng/mL higher alpha-fetoprotein were observed in patients with liver cirrhosis than in non-cirrhotic patients. All HCC prediction model scores differed between the groups, suggesting a significantly higher risk of HCC in the liver cirrhosis group (Table 1).

### 3.2. Antiviral Responses and Changes in Fibrosis Indexes

We analyzed the laboratory data for up to 360 weeks. In the full analysis set, the rates of virological response (HBV DNA < 20 IU/mL) and alanine aminotransferase normalization (male < 41 U/L and female < 33 U/L) were 95.7% and 90.7%, respectively (Table S2). In one patient, loss of HBsAg occurred during follow-up. Antiviral resistance to BSV was not observed.

The cumulative incidence of virologic response was significantly higher in patients with liver cirrhosis than in those without it in the full analysis set (*p* = 0.008) during 360 weeks of follow-up (Figure S2). The cumulative incidences of biochemical responses did not differ between the groups (*p* = 0.133) (Figure S2).

Fibrosis indices, such as APRI and Fib-4, significantly improved during 360 weeks of BSV therapy in CHB patients with and without liver cirrhosis (Table S3). At all time points, the Fib-4 and APRI scores were higher in patients with liver cirrhosis than in those without cirrhosis (*p* < 0.001) (Table S3).

### 3.3. Incidence of HCC and Characteristics of the Patients who Developed HCCs

During the follow-up period of 8 years, HCC developed in five patients. Among them, four patients were male and four were HBeAg-negative. Among the patients who developed HCC, one patient did not have liver cirrhosis (1/139, 0.7%) at baseline, while four had liver cirrhosis (4/49, 8.2%). The risk scores tended to be high in patients who developed HCC; four and five patients were in the high-risk group as assessed by the GAG-HCC and REACH-B models, respectively, which were derived from patients with CHB who did not receive antiviral therapy (Table S4).

### 3.4. Identification of Risk Factors on the HCC Development

**Figure 1:**
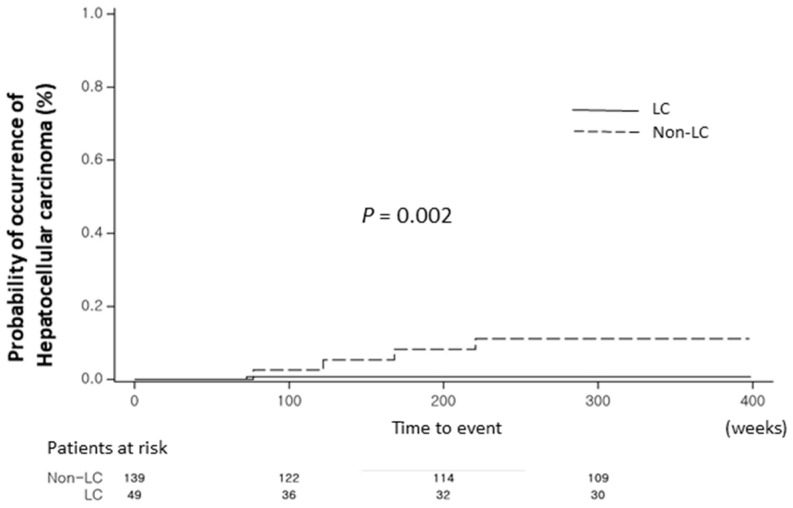
Cumulative incidence of HCC according to the presence of cirrhosis at baseline. *Abbreviations:* HCC, hepatocellular carcinoma.

To investigate the risk factors associated with the development of HCC in patients with CHB, a Cox proportional hazard regression model was used for univariable and multivariable analyses; the presence of liver cirrhosis was a significant factor for HCC according to univariable and multivariable analyses (*p* = 0.02 and *p* = 0.03, respectively) (Table 2). The presence of liver cirrhosis increased the HCC risk by up to 11.125-fold compared to the risk in those without liver cirrhosis (HR, 11.125; 95% CI, 1.232–100.447) after adjusting for age. Figure 1 shows the time to HCC development in patients with or without liver cirrhosis, demonstrating a significant difference between the groups (*p* = 0.002).

### 3.5. The Influence of BSV Treatment on the Prediction of HCCs

**Figure 2:**
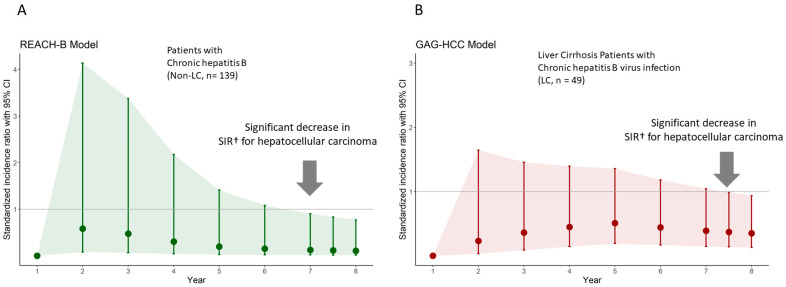
Changes in standardized incidence ratio for hepatocellular carcinoma in chronically hepatitis B virus-infected patients receiving besifovir therapy. (**A**) Patients without liver cirrhosis. (**B**) Patients with liver cirrhosis. † calculated based on the incidence of HCC compared with the predictive numbers of HCC using the each prediction model. *Abbreviations*: REACH-B, risk estimation for HCC in chronic hepatitis B [4]; GAG-HCC, guide with age, gender, HBV DNA, core promotor mutation, and cirrhosis [2]; CI, confidence interval; LC, liver cirrhosis; *n*, number; SIR, standardized incidence ratio.

In the present study, patients were treatment-naïve before enrollment and were treated with BSV for up to 8 years, including the first year of TDF for half of the patients. In patients without underlying liver cirrhosis at baseline (139/188, 73.9%), we applied the REACH-B model to predict HCC, which was devised for CHB patients without liver cirrhosis. The SIR was calculated for up to 8 years using the number of HCC cases from observation over prediction. At 7 years, the SIR was 0.128 (95% CI, 0.018–0.905; *p* = 0.039), suggesting a significant reduction in the development of HCC cases (*n* = 1) under BSV therapy compared with the numbers predicted by the REACH-B model (*n* = 7.84). At eight years, the SIR was 0.109 (95% CI, 0.015–0.773; *p* = 0.027), suggesting a further reduction in the risk in the development of HCC (Table 3) (Figure 2A).

In patients with liver cirrhosis at baseline (49/188, 26.1%), the SIR was calculated based on the GAG-HCC model. The mean GAG-HCC score of patients with liver cirrhosis was 113.15, suggesting a high-risk group. When 22% of HCC prediction at 7.5 years was applied, 10.78 cases of HCC were predicted, while four cases of HCC were observed in this study (SIR, 0.371; 95% CI, 0.139–0.989; *p* = 0.047), indicating a significant reduction in HCC incidence in patients with liver cirrhosis (Table 4) (Figure 2B).

### 3.6. Risk Scores on HCC Development and Performance of Prediction Models

We evaluated the performance of HCC prediction models using the Cox proportional hazards regression model in patients undergoing BSV treatment. Most of models predicted HCC development well, with statistical significance (*p* < 0.05) except PAGE-B (Table 5). When we compared the performance of the AUROCs, the HCC-RESCUE model showed a higher AUROC value (0.924) than the PAGE-B (0.698), mPAGE-B (0.771), REAL-B (0.866), CAMD (0.912), and AASL (0.909) models (Figure S3; Table 5); however, there were no statistically significant differences (Table S5).

## 4. Discussion

BSV is a currently approved acyclic nucleotide phosphonate for the treatment of CHB in Korea [12]. The efficacy of BSV was comparable to that of ETV or TDF in phase 2 and phase 3 studies, without the development of antiviral resistance [12,21]. As mentioned above, the safety profile of BSV is better than that of TDF in terms of renal and bone toxicity. Hence, long-term treatment with BSV for CHB is considered safe and is expected to reduce complications of chronic liver diseases, such as the progression of liver fibrosis or cirrhosis and the development of HCC [12]. In this regard, we evaluated the incidence of HCC in chronically HBV-infected patients with BSV who participated in the phase 3 trial.

To compare the incidence of HCC in CHB patients with and without BSV therapy, we adopted the REACH-B model, which was devised to predict HCC in untreated CHB patients without cirrhosis [4]. SIR, a ratio comparing observed incidence over predicted incidence, was significantly reduced to 0.128 at 7 years and 0.109 at 8 years of BSV treatment. Only one patient who developed HCC among non-cirrhotic patients had a high-risk score for REACH-B and other risk models. After 2 years, no more CHB patients without cirrhosis developed HCC for 8 years. Previously, Kim et al. reported a reduced SIR of HCC to 0.40 with TDF therapy in the TDF cohort under clinical trials [8]. Here, the SIR after BSV therapy was lower than that after TDF therapy in a previous study, although a head-to-head comparison could not be performed.

For patients with cirrhosis, we adopted the GAG-HCC model because the REACH-B model does not support the prediction of HCC in cirrhotics [2,4]. The derivative cohort of the GAG-HCC model comprised cirrhotic and non-cirrhotic CHB patients who did not receive antiviral therapy. Hence, we estimated the incidence of HCC using the prediction graphs from the GAG-HCC model in untreated CHB patients with liver cirrhosis, and approximately 22% of the incidence at year 7.5 was predicted [2]. Afterwards, we compared it with the observed incidence; SIR was 0.371, which suggests a significant reduction in HCC incidence with BSV therapy in patients with liver cirrhosis. These findings suggest that halting the carcinogenetic process by antiviral therapy is possible [8,9,22], although the time might be slightly more delayed in patients with liver cirrhosis than in non-cirrhotic patients.

To predict the long-term outcomes after BSV therapy, we applied our data to existing HCC prediction models, such as PAGE-B [5], mPAGE [17], CAMD [18], HCC-RESCUE [6], REAL-B [19], and AASL [20]. These models were developed to predict HCC in patients with chronic HBV infection under ETV or TDF therapy. When the models were applied to the patients receiving BSV therapy, we found that the performance of these models was good to excellent in this BSV cohort. In particular, the AUROC was higher for HCC-RESCUE (0.924), CAMD (0.912), and AASL (0.909), although statistical differences were not observed. HCC-RESCUE was derived from patients receiving ETV [6], and AASL and CAMD were derived from a cohort in which 63.7% and 96.3% of patients were treated with ETV, respectively [17,19]. It is thought that the effect of HCC risk reduction by BSV therapy may be similar to that of ETV, although further comparative studies of BSV vs. ETV or TDF are needed.

To evaluate the risk factors for HCC in patients with chronic HBV infection who received BSV, we performed a multivariable analysis. As a result, only the presence of liver cirrhosis was a significant factor for the development of HCC in this cohort, which is consistent with previous studies on antiviral therapy [6,18,19,20]. As we did not measure liver stiffness at baseline, liver cirrhosis was defined using clinical and histological criteria. However, liver stiffness measurement, which is a noninvasive method to diagnose liver cirrhosis, could be a more convenient tool to define the presence of liver cirrhosis and stratify the degree of liver fibrosis [23,24]. Instead, we assessed Fib-4 and APRI for the evaluation of the degree of liver fibrosis at baseline as well as during follow-up and demonstrated its significant improvement during 360 weeks of therapy [25,26,27]. We believe that the reduction in fibrotic burden and intrahepatic necroinflammation caused by suppression of viral replication via long-term BSV therapy would be related to a reduced incidence of HCC.

Recently, Murata et al. demonstrated that nucleotide analogues have additional effects on the induction of interferon-λ3, which inhibits HBsAg production and carcinogenesis in patients with CHB [28,29]. On the contrary, interferon-λ3 did not increase in patients treated with nucleoside analogues such as lamivudine or ETV. Current clinical data favoring a lower incidence of HCC in patients treated with TDF compared with those with ETV may be associated with such findings. BSV belongs to nucleotide analogues but not to nucleoside analogues, so interferon-λ3 may have been induced although further demonstration is necessary. Additionally, it was shown that acyclic nucleotide phosphonates inhibit Akt phosphorylation in the mammalian target of rapamycin pathway, leading to anti-HCC effects [28,30]. It is thought that the decreased incidence of HCC by BSV may be explained by these mechanisms further.

This study had several limitations. First, the number of patients in this cohort was small for the assessment of reduced risk of HCC by BSV because this study was originally designed as a phase 3 trial for the drug approval of an antiviral agent. Hence, we adopted the concept of standardized incidence ratio; predictive values of GAG-HCC and observed incidence were compared. However, this is not a real comparison but an assumption based on prediction. So, we need to validate the result in large real-life cohorts in the near future. Second, we did not include a control group that was not being treated. However, it would be unethical to enroll a control arm without antiviral therapy because the benefits of nucleos(t)ide analogues are well known. Third, the availability of BSV is limited outside Korea. Hence, the present result may not be applicable in countries where BSV is not available. Fourth, a comparison of BSV with other treatments, such as ETV or TDF, was not performed. As the antiviral efficacy of BSV is comparable to that of ETV or TDF, long-term outcomes such as HCC risk reduction might be similar. Nevertheless, such a comparison should be performed soon to guide clinicians in making proper medical decisions regarding the selection of antiviral agents. Finally, an independent HCC prediction model for patients with BSV has not been developed because of the limited number of patients. After including more patients from a real-world cohort, we are now considering the establishment of a new model.

## 5. Conclusions

In conclusion, to our knowledge, this is the first study to report a decreased incidence of HCC after BSV therapy, although it did not include a direct comparison between treatment and no treatment. We also compared HCC prediction models suitable for patients with chronic HBV infection who were treated with BSV. A best-fit prediction model for these patients is warranted.

## Figures and Tables

**Table 1:** Patients’ baseline characteristics.

Variable ^§^		All Patients (*n* = 188)	Non-Cirrhotic Patients (*n* = 139)	Liver Cirrhosis (*n* = 49)	*p*-Value
Male sex, *n* (%)		120 (63.83)	87 (62.59)	33 (67.35)	0.551
Age (years)		45.2 ± 10.96	43.5 ±10.80	50.2 ± 9.94	<0.001
Positive HBeAg, *n* (%)		112 (59.57)	87 (62.59)	25 (51.02)	0.156
HBV DNA (log IU/mL)		6.40 ± 1.63	6.51 ± 1.63	6.08 ± 1.60	0.075
ALT (U/L)		115.65 ± 120.46	117.63 ± 126.80	110.04 ± 101.30	0.767
AST (U/L)		77.91 ± 68.90	73.15 ± 64.66	91.43 ± 78.89	0.386
DM, *n* (%)		35 (18.5)	18 (13.0)	17 (34.7)	0.001
Platelet (10^3^/μL)		189.77 ± 67.07	202.39 ± 52.39	153.98 ± 88.69	<0.001
Bilirubin (mg/dL)		0.67 ± 0.33	0.66 ± 0.35	0.69 ± 0.29	0.375
Albumin (g/dL)		4.25 ± 0.31	4.28 ± 0.30	4.17 ± 0.32	0.045
AFP (ng/mL)		16.80 ± 59.04	9.96 ± 18.83	36.19 ± 109.73	<0.001
APRI		1.30 ± 1.47	1.05 ± 0.94	2.04 ± 2.00	0.003
Fib-4		2.06 ± 1.79	1.57 ± 0.76	3.47 ± 2.83	<0.001
Prognostic scores	GAG-HCC	84.25 ± 20.99	74.07 ± 12.41	113.15 ± 11.03	<0.001
	REACH-B	10.88 ± 2.16	10.65 ± 2.19	11.55 ± 1.94	0.012
	PAGE-B	11.63 ± 4.98	10.68 ± 4.99	14.39 ± 3.88	<0.001
	mPAGE-B	8.97 ± 3.11	8.33 ± 3.08	10.78 ± 2.41	<0.001
	CAMD	7.78 ± 5.08	5.50 ± 3.68	14.24 ± 1.91	<0.001
	HCC-RESCUE	60.79 ± 17.86	52.86 ± 12.47	83.29 ± 9.81	<0.001
	REAL-B	3.90 ± 2.07	3.01 ± 1.45	6.43 ± 1.35	<0.001
	AASL	8.88 ± 5.89	5.69 ± 2.52	17.92 ± 2.00	<0.001

^§^ Data with continuous variables are expressed as mean ± standard deviation and data with categorical variables are expressed as numbers (%). *Abbreviations:* HBV, hepatitis B virus; ALT, alanine aminotransferase; AST, aspartate aminotransferase; DM, diabetes mellitus; AFP, alpha-fetoprotein; REACH-B, risk estimation for hepatocellular carcinoma in chronic hepatitis B [4]; PAGE, patient’s age-gender-platelets score [5]; mPAGE-B, modified PAGE-B [17]; GAG-HCC, guide with age, gender, HBV DNA, core promotor mutation, and cirrhosis; REAL-B [2], Real-World Effectiveness from the Asia Pacific Rim Liver Consortium for HBV [19]; HCC-RESCUE, hepatocellular carcinoma-risk estimating score in chronic hepatitis B under entecavir [6]; CAMD, cirrhosis, age, male sex, diabetes mellitus [18]; AASL, age, albumin, sex, liver cirrhosis [20].

**Table 2:** Univariable and multivariable analyses using the Cox proportional hazard regression model to identify risk factors for HCC development.

	Univariable Analysis				Multivariable Analysis			
	HR	Lower	Upper	*p*-Value	HR	Lower	Upper	*p*-Value
Cirrhosis	13.629	1.523	121.957	0.020	11.125	1.232	100.447	0.032
Age (≥50 years)	7.368	0.824	65.913	0.074	5.579	0.618	50.366	0.126
Male	2.085	0.233	18.655	0.511				
DM	0.997	0.111	8.923	0.998				
HBeAg positive	5.956	0.666	53.295	0.111				
HBV DNA	0.658	0.395	1.098	0.109				
Platelet	1.000	0.986	1.014	0.984				
Albumin	1.627	0.090	29.476	0.742				
AFP	0.996	0.965	1.029	0.822				
AST	0.999	0.984	1.013	0.856				
ALT	0.990	0.968	1.011	0.339				

*Abbreviations:* HCC, hepatocellular carcinoma; HR, hazard ratio; DM, diabetes mellitus; HBeAg, Hepatitis E antigen; HBV, hepatitis B virus; AFP, alpha-fetoprotein; AST, aspartate aminotransferase; ALT, alanine aminotransferase.

**Table 3:** Standardized incidence ratio for HCC using the REACH-B model from years 1 to 8 of besifovir therapy in non-cirrhotic patients (*n* = 139).

Year	Observation (Cumulative No. of Cases)	Prediction	SIR (Observation/ Prediction)	95% CI Lower Limit	95% CI Upper Limit	*p*-Value
1	0	0.73	0	0	-	1.000
2	1	1.72	0.583	0.082	4.135	0.589
3	1	2.10	0.475	0.067	3.375	0.457
4	1	3.26	0.306	0.043	2.175	0.237
5	1	5.01	0.199	0.028	1.416	0.107
6	1	6.55	0.153	0.022	1.084	0.060
7	1	7.84	0.128	0.018	0.905	0.039
7.5	1	8.51	0.118	0.017	0.834	0.032
8	1	9.18	0.109	0.015	0.773	0.027

*Abbreviations:* HCC, hepatocellular carcinoma; REACH-B, risk estimation for hepatocellular carcinoma in chronic hepatitis B [4]; SIR, standardized incidence ratio; CI, confidence interval.

**Table 4:** Standardized incidence ratio for HCC using the GAG-HCC model from years 1 to 8 of besifovir therapy in patients with cirrhosis (*n* = 49).

Year	Observation (Cumulative no. of Cases)	Prediction	SIR (Observation/ Prediction)	95% CI Lower Limit	95% CI Upper Limit	*p*-Value
1	0	3.14	0	0	.	1
2	1	4.31	0.232	0.033	1.646	0.144
3	2	5.49	0.364	0.091	1.457	0.153
4	3	6.66	0.450	0.145	1.396	0.167
5	4	7.84	0.510	0.192	1.359	0.178
6	4	9.02	0.444	0.167	1.182	0.104
7	4	10.19	0.392	0.147	1.046	0.061
7.5	4	10.78	0.371	0.139	0.989	0.047
8	4	11.37	0.352	0.132	0.938	0.037

*Abbreviations:* HCC, hepatocellular carcinoma; GAG-HCC; guide with age, gender, HBV DNA, core promoter mutation, and cirrhosis [2]; SIR, standardized incidence ratio; CI, confidence interval.

**Table 5:** Performance of HCC prediction models in patients with chronic hepatitis B virus infection receiving besifovir therapy.

	Cox’s Proportional Hazard Regression Model				AUROC		
	HR	Lower	Upper	*p*-Value	Value	Lower	Upper
PAGE-B	1.195	0.957	1.491	0.116	0.698	0.385	1.000
mPAGE-B	1.480	1.006	2.177	0.047	0.771	0.508	1.000
REAL-B	1.872	1.154	3.037	0.011	0.866	0.806	0.925
HCC-RESCUE	1.127	1.041	1.221	0.003	0.924	0.851	0.998
CAMD	1.573	1.132	2.188	0.007	0.912	0.832	0.991
AASL	1.345	1.068	1.693	0.012	0.909	0.821	0.998

*Abbreviations:* HCC, hepatocellular carcinoma; PAGE, patient’s age–gender–platelet score [5]; mPAGE-B, modified PAGE-B [17]; REAL-B, Real-World Effectiveness from the Asia Pacific Rim Liver Consortium for HBV [19]; HCC-RESCUE, hepatocellular carcinoma-risk estimating score in chronic hepatitis B under entecavir [6]; CAMD, cirrhosis, age, male sex, diabetes mellitus [18]; AASL, age, albumin, sex, liver cirrhosis [20].

## Data Availability

The datasets used and/or analyzed during the current study are available from the corresponding author on reasonable request.

## Supplementary Materials

The following supporting information can be downloaded at: https://www.mdpi.com/article/10.3390/cancers16050887/s1, Figure S1: Study flow; Figure S2: Virological and biochemical responses in patients with and without liver cirrhosis during besifovir treatment in the full analysis set. (a) Virological responses. (b) Biochemical responses; Figure S3: ROC curves of risk prediction models for hepatocellular carcinoma. (a) PAGE-B, (b) mPAGE-B, (c) CAMD, (d) HCC-RESCUE, (e) REAL-B, (f) AASL; Table S1: Comparison of HCC prediction models in patients with chronic hepatitis B virus infection; Table S2: Virological, biochemical, and serologic responses over time in the study population; Table S3: Changes in APRI and FIB4 score according to cirrhosis status during besifovir therapy; Table S4: Characteristics of the patients who developed HCCs during besifovir treatment; Table S5: Comparison of AUROCs between different prediction models for HCC in patients with chronic HBV infection receiving besifovir therapy.
