# Supplementary material for: Reduced Risk of Hepatocellular Carcinoma in Patients with Chronic Hepatitis B Receiving Long-Term Besifovir Therapy
Source: Cancers (Basel). 2024 Feb 22;16(5):887. doi: 10.3390/cancers16050887 (PMC10930911; doi:10.3390/cancers16050887)
Supplement: Supplementary file 1 [file cancers-16-00887-s001.zip › cancers-2813104-supplementary.pdf]

Suppl. Table S1. Comparison of HCC prediction models in patients with chronic hepatitis B virus infection†

Model (year)	Subjects (derivation)	Country	Etiology	Treatment (%)	Antiviral agent	Cirrhosis (%)	Year of Prediction	Variables	Low/intermediate/High
GAG-HCC [2] (2009)	820	Hong Kong	CHB, LC	0%	None	15.1%	10	Age, male, BCP, LC, HBV DNA	<100/–/ ≥100
REACH-B [4] (2011)	3,584	Taiwan	CHB	0%	None	0%	10	Age, male, ALT, HBeAg, HBV, DNA	0–5/6–11/12–18
PAGE-B [5] (2015)	1,325	Europe	CHB, LC	100%	ETV, TDF (Hx of other NA 33%)	20%	5	Age, male, PLT	<9/10–17/≥18
mPAGE-B [17] (2018)	2001	Korea	CHB, LC	100%	ETV, TDF	19.1%	5	Age, male, PLT, albumin	≤8/9–12/ ≥13
CAMD [18] (2018)	23,851	Taiwan (derivation)	CHB, LC	100%	ETV (96.3%), TDF (3.7%)	26.5%	3	Age, LC, male, DM	<8/8–13/>13
HCC-RESCUE [6] (2016)	990	Korea	CHB, LC	100%	ETV (100%)	39%	5	Age, male, LC	≤64/65–84/ ≥85
REAL-B [19] (2020)	5,365	Taiwan, US & AP region	CHB, LC	100%	LAM, ADV, LDT, ETV, TDF	20.2%	10	Age, male, alcohol, DM, LC, PLT,	0–3/4–7/8–13

								AFP	
AASL [20] (2019)	944	Korea	CHB, LC	100%	ETV (63.7%), TDF (36.3%)	39.3%	5	Age, albumin, male, LC	≤5/6–19/≥20

† Models requiring liver stiffness measures are excluded (e.g., LS-HCC, mREACH-B, and SAGE/CAGE). Additionally, HCC prediction models developed not only from CHB but also from various etiologies of chronic liver diseases were excluded (e.g. aMAP. THRI).

Abbreviations: HCC, hepatocellular carcinoma; GAG-HCC, guide with age, gender, HBV DNA, core promotor mutation, and cirrhosis [2]; REACH-B, risk estimation for hepatocellular carcinoma in chronic hepatitis B [4]; PAGE-B, patient's age-gender-platelets score [5]; mPAGE-B, modified PAGE-B [17]; HCC-RESCUE, hepatocellular carcinoma-risk estimating score in chronic hepatitis B under entecavir [6]; CAMD, cirrhosis, age, male sex, diabetes mellitus [18]; AASL, age, albumin, sex, liver cirrhosis [20]; REAL-B, Real-World Effectiveness from the Asia Pacific Rim Liver Consortium for HBV [19]; CHB, chronic hepatitis B; LC, liver cirrhosis; NA, not attributable; ETV, entecavir; TDF, tenofovir disoproxil fumarate; LAM, lamivudine; ADV, adefovir; LDT, telbivudine; BCP, basal core promotor mutation; ALT, alanine aminotransferase; HBeAg, hepatitis B e antigen; PLT, platelet; AFP, alpha-fetoprotein

Suppl. Table S2. Virological, biochemical, and serologic responses over time in the study population

Frequency§ n (%)	Week 48	Week 96	Week 144	Week 192	Week 240	Week 288	Week 336	Week 360
Full Analysis Set n (%)	188	169	156	151	147	146	142	140
HBV DNA <69 IU/mL	153 (81.4)	146 (86.4)	140 (89.7)	140 (92.7)	137 (93.2)	140 (95.9)	135 (95.1)	136 (97.1)
HBV DNA <20 IU/mL	122 (64.9)	134 (79.3)	129 (82.7)	132 (87.4)	130 (88.4)	137 (93.8)	132 (93.0)	134 (95.7)
ALT normalization†	138 (73.4)	136 (80.5)	132 (84.6)	137 (90.7)	137 (93.2)	133 (91.1)	128 (90.1)	127 (90.7)
HBeAg seroclearance‡	8 (7.8)	20 (20.8)	23 (25.3)	30 (33.7)	36 (41.9)	38 (44.7)	39 (46.4)	n/c
HBsAg seroclearance	1 (0.53)	1 (0.59)	1 (0.64)	1 (0.66)	1 (0.68)	1 (0.68)	1 (0.70)	n/c

§Data are expressed as numbers (%), and missing data were not counted.

† ALT normalization was defined as <41 U/L for males and <33 U/L for females according to laboratory reference values.

‡ Assessed in patients with positive hepatitis B e antigen at baseline

Abbreviations: HBV, hepatitis B virus; ALT, alanine aminotransferase; HBeAg, hepatitis B e antigen; HBsAg, hepatitis B surface antigen; n/c, not collected

Suppl. Table S3. Changes in APRI and FIB4 score according to cirrhosis status during besifovir therapy

			APRI			FIB-4		
**Week**	**Cirrhosis**	**N**	**Mean**	**STD**	***P*-value§**	**Mean**	**STD**	***P*-value†**
Baseline	N	139	1.046	0.938	0.003	1.566	0.760	<0.001
	Y	49	2.038	2.002		3.474	2.832	
48	N	131	0.447	0.325	<0.001	1.275	0.705	<0.001
	Y	42	0.763	0.809		2.299	1.924	
96	N	124	0.364	0.253	<0.001	1.136	0.617	<0.001
	Y	36	0.599	0.386		2.005	1.075	
144	N	119	0.346	0.248	<0.001	1.108	0.574	<0.001
	Y	34	0.569	0.521		1.930	1.072	
192	N	116	0.311	0.197	0.001	1.059	0.576	<0.001
	Y	34	0.484	0.311		1.767	0.834	
240	N	113	0.307	0.162	<0.001	1.078	0.537	<0.001
	Y	34	0.411	0.198		1.733	0.993	
288	N	109	0.311	0.175	<0.001	1.100	0.838	<0.001
	Y	33	0.447	0.290		1.689	0.811	
336	N	109	0.304	0.199	<0.001	1.051	0.534	<0.001
	Y	33	0.404	0.226		1.656	0.914	
360	N	105	0.329	0.345	<0.001	1.069	0.572	<0.001
	Y	32	0.410	0.217		1.763	1.229	
Overall comparison between the groups (LC vs. non-LC) §					<0.001			<0.001
Overall comparisons within the group (LC or non-LC) §								
0–360	N	139			<0.001			<0.001
	Y	49			<0.001			<0.001

†Comparison between the groups at each time point using Wilcoxon rank-test or independent t-test according to the normality of the data

[§]Comparison of data from 48 to 360 weeks within the group using linear mixed models

Abbreviations: APRI, aspartate aminotransferase to platelet ratio index; Fib-4, fibrosis 4; STD, standard deviation; N, no; Y, yes

Suppl. Table S4. Characteristics of the patients who developed HCCs during besifovir treatment

No	Sex	Age	Time to the event (week)	Year of HCC Dx	HBeAg	PLT	HBV DNA (log IU/mL)	ALT (U/L)	FIB4	APRI	LC†	GAG	REACH-B	PAGE	mPAGE	CAMD	RESCUE	REAL-B	AASL
1	M	61	72.6	2	Neg	189	6.76	43	2.95	0.79	N	95.3	13	20	14	11	76	6	12
2	F	67	76.9	2	Neg	208	6.13	120	2.79	1.43	Y	118.4	12	8	11	19	90	6	16
3	M	62	122	3	Neg	166	5.93	68	3.40	1.13	Y	126.8	15	20	14	22	100	7	18
4	M	56	168.1	4	Neg	106	3.86	63	3.86	1.37	Y	114.6	9	18	13	20	94	7	16
5	M	46	220.7	5	Pos	283	7.27	62	1.55	0.66	Y	114.8	13	10	7	18	84	6	14

†Liver cirrhosis was defined by histological and clinical criteria: **(1)** microscopic findings of liver cirrhosis (F5 or F6) by Ishak-Knodell scoring system; **(2)** ultrasonography findings suggestive of cirrhosis; or **(3)** biomarker for liver fibrosis predictive of liver cirrhosis (≥ FIB-4 score 3.6).

Abbreviations: HCC, hepatocellular carcinoma; No, patient number; HBeAg, hepatitis B virus e antigen; PLT, platelet; ALT, alanine aminotransferase; Fib-4, fibrosis 4; APRI, aspartate aminotransferase to platelet ratio index; LC, liver cirrhosis; Dx, diagnosis; REACH-B, risk estimation for hepatocellular carcinoma in chronic hepatitis B [4]; PAGE-B, patient's age-gender-platelets score [5]; mPAGE-B, modified PAGE-B [17]; GAG-HCC, guide with age, gender, HBV DNA, core promotor mutation, and cirrhosis [2]; REAL-B, Real-World Effectiveness from the Asia Pacific Rim Liver Consortium for HBV [19]; HCC-RESCUE, hepatocellular carcinoma-risk estimating score in chronic hepatitis B under entecavir [6]; CAMD, cirrhosis, age, male sex, diabetes mellitus [18]; AASL, age, albumin, sex, liver cirrhosis [20]; M, male; F, female; Pos, positive; Neg, negative; Y, yes; N, no

Suppl. Table S5. Comparison of AUROCs between different prediction models for HCC in patients with chronic HBV infection receiving besifovir therapy

Comparison (*P*-value†)	PAGE	mPAGE	REAL-B	HCC-RESCUE	CAMD
mPAGE	0.499				
REAL-B	0.256	0.448			
HCC-RESCUE	0.176	0.256	0.044		
CAMD	0.208	0.311	0.146	0.185	
AASL	0.217	0.331	0.210	0.223	0.798

†Comparison using the DeLong test.

Abbreviations: AUROC, area under the receiver operating curve; HCC, hepatocellular carcinoma; PAGE-B, patient's age-gender-platelets score [5]; mPAGE-B, modified PAGE-B [17]; REAL-B, Real-World Effectiveness from the Asia Pacific Rim Liver Consortium for HBV [19]; HCC-RESCUE, hepatocellular carcinoma-risk estimating score in chronic hepatitis B under entecavir [6]; CAMD, cirrhosis, age, male sex, diabetes mellitus [18]; AASL, age, albumin, sex, liver cirrhosis [20]

Suppl. Figure S1. Study flow.

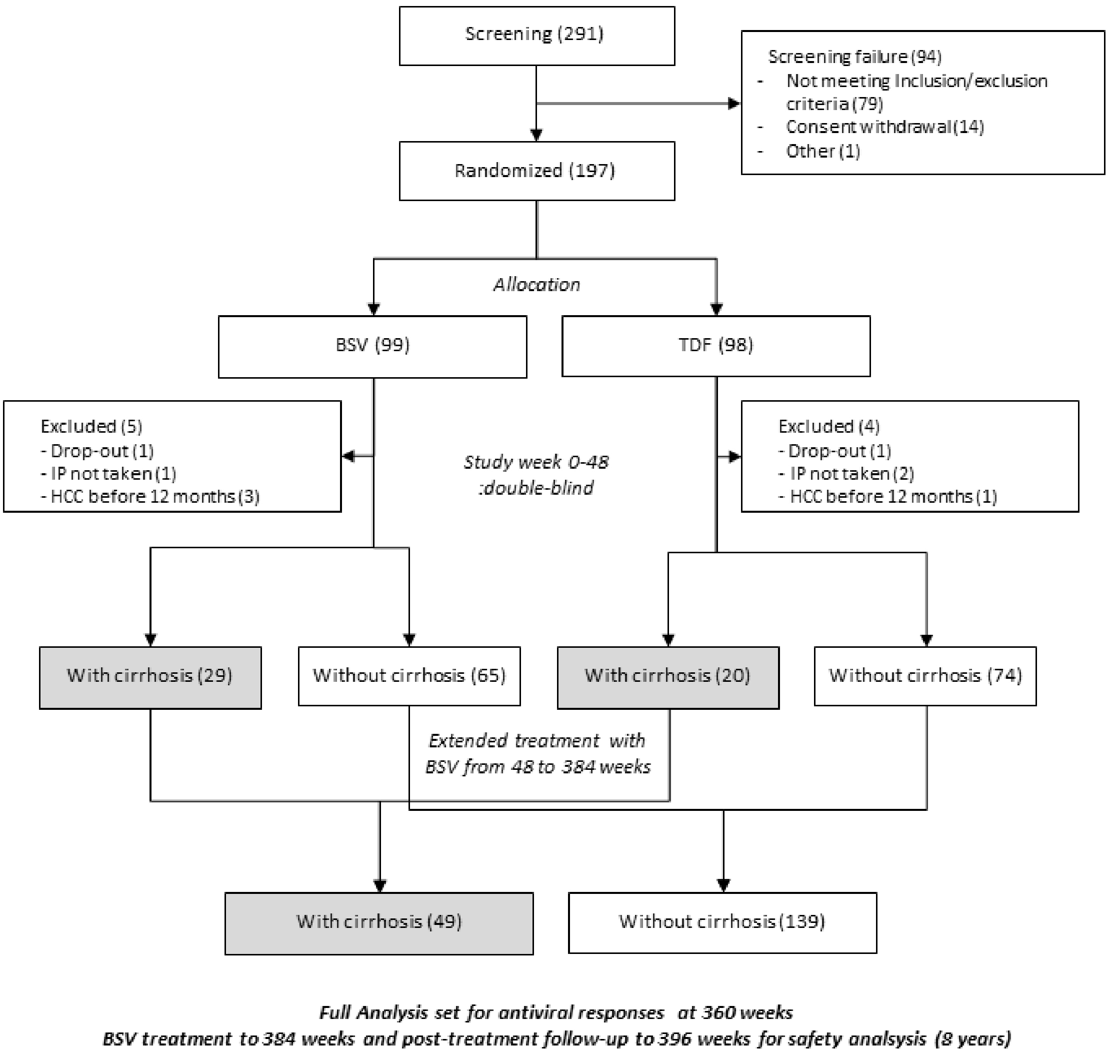

Abbreviation: BSV, besifovir; TDF, tenofovir disoproxil fumarate; IP, investigational product; HCC, hepatocellular carcinoma

Suppl. Figure S2. Virological and biochemical responses in patients with and without liver cirrhosis during besifovir treatment in the full analysis set (a) Virological responses (b) Biochemical responses.

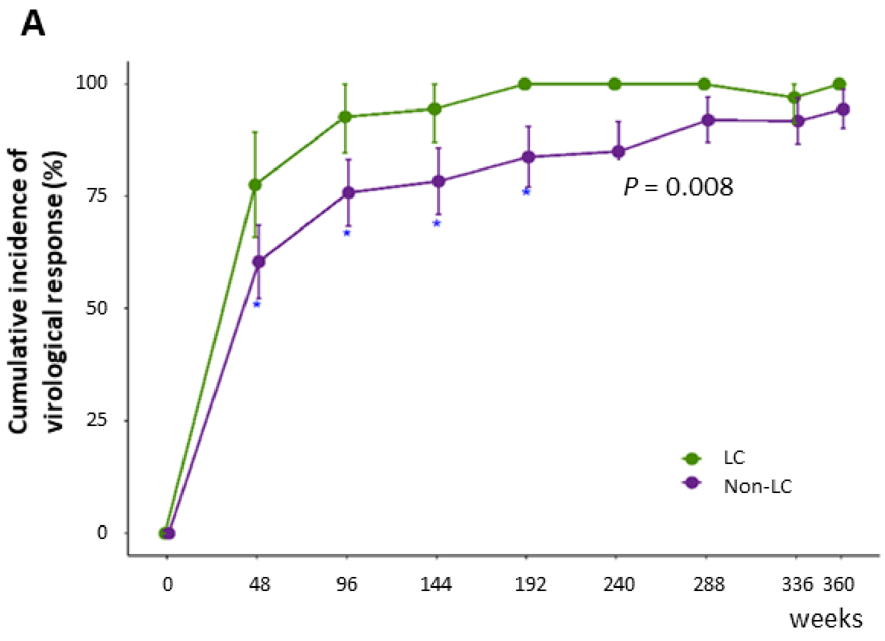

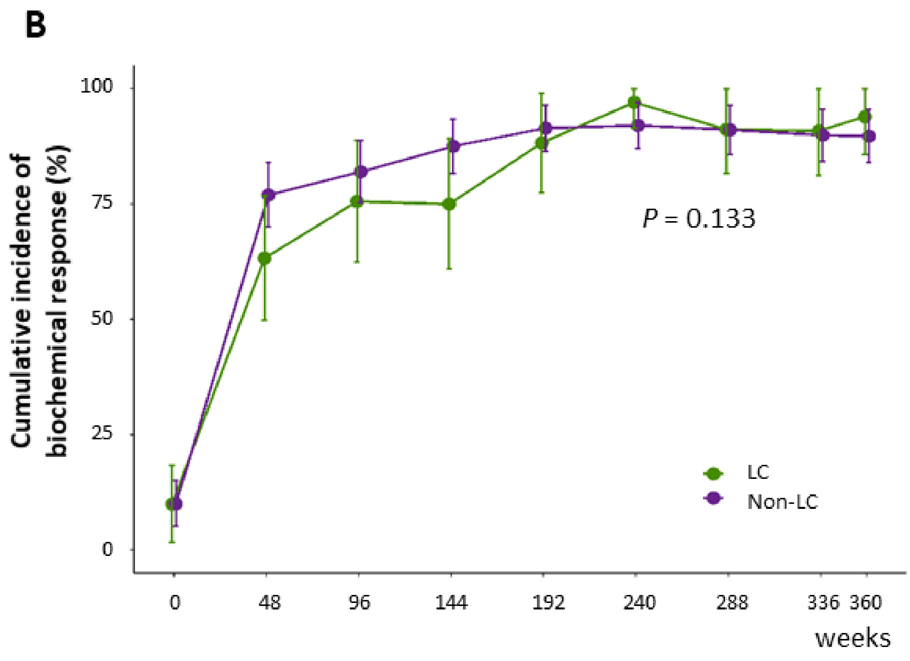

Suppl. Figure S3. ROC curves of risk prediction models for hepatocellular carcinoma (a) PAGE-B, (b) mPAGE-B, (c) CAMD, (d) HCC-RESCUE, (e) REAL-B, (f) AASL.

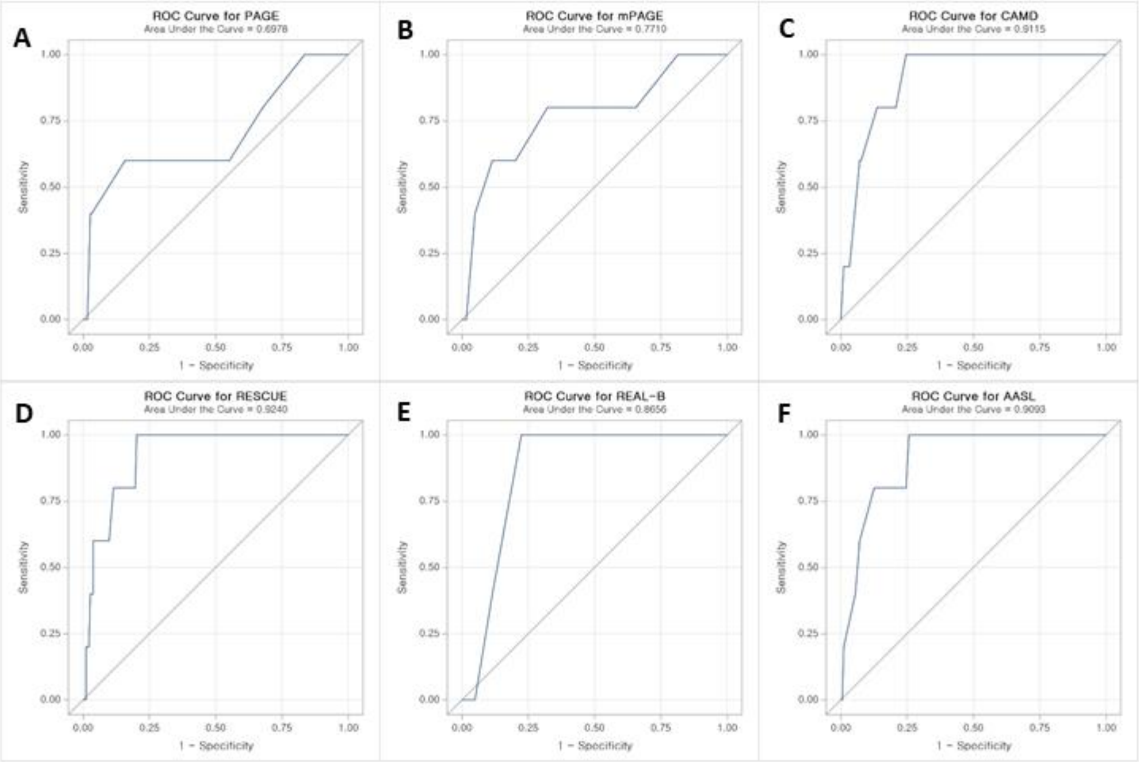

Abbreviations: ROC, receiver operating curve; PAGE-B, patient's age-gender-platelets score [5]; mPAGE-B, modified PAGE-B [17]; CAMD, cirrhosis, age, male sex, diabetes mellitus [18]; HCC-RESCUE, hepatocellular carcinoma-risk estimating score in chronic hepatitis B under entecavir [6]; REAL-B, Real-World Effectiveness from the Asia Pacific Rim Liver Consortium for HBV [19]; AASL, age, albumin, sex, liver cirrhosis [20]; US, United States
